# Human multipotent mesenchymal stem cells improve healing after collagenase tendon injury in the rat

**DOI:** 10.1186/1475-925X-13-42

**Published:** 2014-04-09

**Authors:** Lucia Machova Urdzikova, Radek Sedlacek, Tomas Suchy, Takashi Amemori, Jiri Ruzicka, Petr Lesny, Vojtech Havlas, Eva Sykova, Pavla Jendelova

**Affiliations:** 1 Institute of Experimental Medicine, Academy of Sciences of the Czech Republic, Prague, Czech Republic; 2 Department of Neuroscience, Charles University, Second Faculty of Medicine, Prague, Czech Republic; 3 Department of Orthopedics and Traumatology, Charles University, 2nd Faculty of Medicine, Prague, Czech Republic; 4 Laboratory of Biomechanics, Department of Mechanics, Biomechanics and Mechatronics, Faculty of Mechanical Engineering, Czech Technical University in Prague, Prague, Czech Republic

**Keywords:** Achilles tendon, Mesenchymal stromal cells, Osteogenesis

## Abstract

**Background:**

Mesenchymal stromal cells attract much interest in tissue regeneration because of their capacity to differentiate into mesodermal origin cells, their paracrine properties and their possible use in autologous transplantations. The aim of this study was to investigate the safety and reparative potential of implanted human mesenchymal stromal cells (hMSCs), prepared under Good Manufacturing Practice (GMP) conditions utilizing human mixed platelet lysate as a culture supplement, in a collagenase Achilles tendon injury model in rats.

**Methods:**

Eighty-one rats with collagenase-induced injury were divided into two groups. The first group received human mesenchymal stromal cells injected into the site of injury 3 days after lesion induction, while the second group received saline. Biomechanical testing, morphometry and semiquantitative immunohistochemistry of collagens I, II and III, versican and aggrecan, neovascularization, and hMSC survival were performed 2, 4, and 6 weeks after injury.

**Results:**

Human mesenchymal stromal cell-treated rats had a significantly better extracellular matrix structure and a larger amount of collagen I and collagen III. Neovascularization was also increased in hMSC-treated rats 2 and 4 weeks after tendon injury. MTCO2 (Cytochrome c oxidase subunit II) positivity confirmed the presence of hMSCs 2, 4 and 6 weeks after transplantation. Collagen II deposits and alizarin red staining for bone were found in 6 hMSC- and 2 saline-treated tendons 6 weeks after injury. The intensity of anti-versican and anti-aggrecan staining did not differ between the groups.

**Conclusions:**

hMSCs can support tendon healing through better vascularization as well as through larger deposits and better organization of the extracellular matrix. The treatment procedure was found to be safe; however, cartilage and bone formation at the implantation site should be taken into account when planning subsequent *in vivo* and clinical trials on tendinopathy as an expected adverse event.

## Background

Tendon pathologies are a common problem especially in people of working age. The tendon healing process includes the formation of a hematoma, the local infiltration of inflammatory cells, the release of cytokines and growth factors followed by the formation of new extracellular matrix, new blood vessels and finally maturation and organization of the tendon tissue. Various cell-based therapies have been used to treat tendon pathologies in several *in vivo* studies [1-3] and at least one clinical trial (OrthADAPT; ClinicalTrials.gov identifier NCT01687777). Clinically relevant cell-based therapies often utilize adult stem cells, e.g. multipotent mesenchymal stromal cells. Mesenchymal stromal cells of various origins have been implanted directly into a tendon injury or attached to biodegradable scaffolds used to repair the tendon; they have shown an apparent beneficial effect on the healing process [4] and migrated to the injured tendon [5]. It was repeatedly shown that the delivery of mesenchymal stromal cells can create an optimal environment to support tendon tissue regeneration *via* the formation of extracellular matrix, enhanced vascularization, the production of supporting factors, modulation of the immunoresponse and the replacement of damaged cells [4,6].

Our aim was to describe the effect of the xenogeneic transplantation of human multipotent mesenchymal stromal cells (hMSCs) prepared under Good Manufacturing Practice (GMP) conditions on the healing process of rat Achilles tendons and also to identify potential side effects of human mesenchymal cells (tumorigenesis, wrong differentiation pathways) transplanted into a tendon injury to confirm the safety of hMSC treatment.

## Methods

### Cell isolation and cultivation

hMSCs were isolated from the bone marrow of healthy human donors. All procedures for hMSC preparation were performed under GMP in the spin-off company Bioinova, Ltd. (Prague, Czech Republic) and approved by the State Institute for Drug Control of the Czech Republic (SUKL, Czech Republic). The mononuclear fraction containing hMSCs was separated from the bone marrow by gradient centrifugation using 25% Gelofusine (B. Braun, Melsungen, Germany) and seeded on plastic dishes at a concentration of 5 – 10 × 10^6^ cells/75 cm^2^. The cells were expanded in media containing Alpha MEM Eagle without Deoxyribonucleotides, Ribonucleotides and UltraGlutamin (Lonza, Basel, Switzerland) supplemented with 5% mixed allogeneic thrombocyte lysate (Bioinova, Prague, Czech Republic) and 10 μg/ml Gentamicin (Lek Pharmaceuticals, Ljublanja, Slovenia); non-adherent cells were washed out by changing the medium. When the cells reached 80% confluence, they were detached from the surface of the dishes using 1 ml/75 cm^2^ of TrypLE CTS Select™ solution (Gibco, Ca, USA) and re-seeded at a lower density (5 × 10^4^ per square cm). Cells of the second passage were analyzed and used in further experiments. The expression of specific surface markers was assessed using fluorescent-activated cell sorting (FACS) analysis (FACSAria flow cytometer, BD Biosciences, San Diego, USA). The cells were selected for the following markers – the presence of CD105, CD73 and CD90 and the absence of CD45, CD34, CD14 or CD11b, CD79alpha and HLA-DR surface molecules. In order to verify the differentiation potential of the hMSCs, the cells were differentiated into osteogenic, chondrogenic and adipogenic lineages using standard differentiation media as described previously [7]. Cell viability was evaluated according to GMP quality control requirements by using trypan blue staining, and the cultures were tested for the presence of bacterial, fungal and mycoplasma contamination by methods recognized in the European Pharmacopoeia, article 2.7.29 “Validated cell and viability count using trypan dye exclusion method”, article 2.6.1 “Validated cultivational test for sterility” and article 2.6.7 “Mycoplasma detection by validated nucleic acid amplification”. The cells were frozen in aliquots in saline containing 7.5% dimethylsulfoxide (DMSO, Sigma) and 5% albumin and stored in liquid nitrogen at −160°C until use. The freezing and thawing process was validated and provided cells with the required phenotype and constant viability.

For inducing chondrogenesis, cells were harvested, transferred to polypropylene tubes and differentiated into chondrocytes in pellet cultures (250 000/pellet) in serum-free medium containing DMEM, dexamethasone (0,1 μM), hTGF-β1 (10 ng/ml), L-ascorbic acid (0,05 mM), ITS + 1% and Primocin. The tubes were incubated at 37°C in a 5% CO_2_ incubator. The medium was changed twice a week. Chondrogenic pellets were harvested after 20–22 days in culture. The pellets were fixed with 10% formaldehyde, embedded in parrafin blocks and sectioned into 5 μm sections. Samples were than stained with Alcian blue.

To induce adipogenic differentiation the cells were seeded in six-well plates at a density of 10 000 cells/cm^2^. Cells at confluence were treated with medium containing DMEM, FBS 10%, dexamethasone (1 μM), 3-isobutyl-1-methyl-xanthine (0,5 mM), indomethacin (0,1 mM), insulin (10 μg/ml), and Primocin. The medium was changed twice a week. After 12 days the cells were fixed with 10% formaldehyde and stained with Oil Red O.

Osteogenesis was induced by seeding the cells in six-well plates at a density of 3000 cells/cm^2^. Cells were allowed to adhere for 24 hours, and osteogenic medium containing DMEM, FBS 10% dexamethasone (0,1 μM), β-glycerophosphate (10 mM), L-ascorbic acid (0,1 mM) and Primocin was added. The medium was changed twice a week. After 20 days the cells were fixed with 10% formaldehyde and stained with Alizarin red.

### Tendon injury

All animal experiments were approved by the Animal Committee of the Czech Republic and the Animal Care and Use of Animals Committee of the Institute of Experimental Medicine AS CR. Eighty-one adult rats weighing 400 – 440 g were anesthetized with 2.5% isoflurane mixed with air. Tendon injury was induced by the application of collagenase to the middle part of the Achilles tendon, which produces a chemical deterioration of the tendon tissue accompanied by inflammation in the surrounding tissue. Briefly, the rats’ right legs were fixed in position to achieve 90 degrees bending in the *Articulatio talocruralis*. After a skin incision, the Achilles tendon was exposed. Tendon injury was induced by a solution of 0.3 mg collagenase (Sigma) in 25 μl saline injected into the middle part of the Achilles tendons. The left Achilles tendons were left intact. Rats were randomly divided into two groups. Human MSCs (1 million cells in 100 μl saline) were implanted into the centre of the tendon lesion of 41 rats, 3 days after injury by a single injection. Forty rats received 100 μl saline injected in the same regime as the hMSC suspension. Gentamicin (Lek Pharmaceutical, 5 mg/kg i.m.) was given continuously for seven days after tendon injury to prevent post-surgical infection. Immunosuppression was used to prevent the rejection of the cell transplants. Cyclosporin (Sandimmun Neoral, Novartis, 10 mg/kg, p.o.) and methylprednisolone (Solu-Medrol, Pfizer, 2 mg/kg, i.m.) were injected daily, starting one day before cell transplantation. Carprofen (Rimadyl, Pfizer, 1.5 mg/kg, i.m.) was given twice a day as an analgesic, up to 10 days after tendon injury.

### Health condition of the animals and gross inspection of the tendons

Animals were examined for motor performance using the Basso-Beattie-Bresnahan test [8] (routinely used for locomotor evaluation) immediately after awaking from the anaesthesia, before and after hMSC/saline injection and then randomly during the whole survival period. The wounds were regularly checked for any pathological events (inflammation). Each tendon, after isolation and prior to any further biomechanical or histological evaluation, was visually examined to determine whether there were any gross differences between the groups in terms of tendon diameter, the presence of tumors, calcifications, hyperemia, synechies and/or the thickness of the petitendoneal tissues). Evaluations were performed in a blinded fashion by a single investigator.

### Tissue processing

For histological and immunohistochemical evaluations, the rats were euthanized with pentobarbital (Sigma, 100 mg/kg, i.p.) 2, 4 or 6 weeks after tendon injury (10 rats with hMSC treatment and 10 rats with saline at each time-point, Table 1) and transcardially perfused with phosphate buffered saline (PBS), followed by 4% paraformaldehyde in PBS. The Achilles tendons were dissected and immersed in 4% paraformaldehyde at 4°C until further processing.

**Table 1:** Animals

	**2 weeks**	**4 weeks**	**6 weeks**	**Biomechanical study**	**Total**
hMSCs	10	10	10	11	41
Saline	10	10	10	10	40

For biomechanical measurements, the rats were sacrificed with a lethal dose of pentobarbital (120 mg/kg) 4 weeks after cell transplantation (11 rats with hMSC transplantation and 10 rats from the control group). The Achilles tendons of both the left and right legs were removed with the adjacent bone on one side and the muscles on the other side and left in 4% paraformaldehyde until measurements.

### Histological processing

Dissected fixed tendons were transferred into 10% and 20% sucrose. After freezing, the tendons were cryosectioned into longitudinal sections (10 μm thickness), which were labeled sequentially according to the sectioning order. Around 50 sections were collected from each tendon, and a similar depth for each staining was selected. For good reproducibility and comparability, samples from the control and injury groups were stained at the same time under the same conditions. The sections were examined using light and fluorescent microscopy. For all sections, the images were taken using identical light settings. Evaluations were performed in a blinded fashion by a single investigator.

### Histology score

A histology score based on H&E (hematoxylin and eosin) staining was determined 2, 4 and 6 weeks after tendon injury in both groups. Every 8th coronal section was selected, and 6 different histologic parameters were semiquantitatively scored from 1 (severe changes) to 4 (normal) according to the criteria defined in Table 2. Tendon tissues were evaluated for the linearity of their fibre structure, the shape of the tendon cells, the density of the tendon cells, inflammation, hemorrhage, and the thickness of the epitenon, modified from Nixon [9].

**Table 2:** Histology score

**Variable**	**Score and criteria**
Linearity of the fiber structure	1 = no linear areas
	2 = 20-50% linear
	3 = ˃ 50% linear
	4 = linear (normal)
Shape of the tendon cells	1 = predominantly round
	2 = moderately round
	3 = slightly oval
	4 = linear (normal)
Density of the tendon cells	1 = sheets of cells
	2 = moderate increase
	3 = slight increase
	4 = sparse(normal)
Inflammation (leucocyte deposits in the endotenon and epitenon)	1 = severe increase
	2 = moderate increase
	3 = slight increase
	4 = none
Hemorrhage	1 = predominant hemorrhage
	2 = multiply areas in each field
	3 = sparse or patchy
	4 = none
Thickness of the epitenon	1 = massive fibrosis
	2 = 7–15 cells
	3 = 3–6 cells
	4 = 1–2 cells

### Immunohistochemistry

Immunoistochemistry was used for the semiquantitative assessment of collagen I and III, aggrecan and versican, for evaluation of angiogenesis at the site of injury and for the detection of the presence of hMSCs in the tendon tissue. Detection of collagen II was used to confirm cartilage formation at the site of injury. Staining was performed against collagen I (1:500, ABcam), II (1:200, Sigma), III (1:800, ABcam), versican (1:500, Millipore) and aggrecan (1:1000, Millipore). Neovascularisation was assessed by staining using RECA-1 (ABcam, 1:50). Immunofluorescent staining for human mitochondria (anti-Cytochrome c oxidase subunit II antibody, MTCO2, ABcam) was used to identify possible surviving transplanted cells. Antigen-antibody complexes were visualized using secondary antibodies conjugated with Alexa-Fluor 488 or 594 (Molecular Probes).

### Extracellular matrix

Immunohistochemical analysis was used to assess the formation and deposition of collagen type I, collagen type III, aggrecan and versican proteins in every 8th longitudinal section of the tendons. The extent and intensity of the immunoreactions were graded on a scale from 1–4, with a score of 1 indicating the absence of antigen expression, a score of 2 weak and spotted antigen expression, a score of 3 weak, but diffuse antigen expression throughout the entire repaired site and a score of 4 (diffuse and strong antigen expression).

### Angiogenesis

RECA immunostaining was used to evaluate angiogenesis within the Achilles tendon injury. Microscope fields at 40× magnification were centered over the area of Achilles tendon injury. The vessels within this area were counted in a blinded manner, and the number of vessels per mm^2^ was calculated.

### Osteogenesis

Osteogenesis was detected by Alizarin red and Alcian blue staining. Briefly, sections prepared as described above were stained with Alizarin red (Sigma) or Alcian blue (Sigma) for a duration of 20 seconds to 5 minutes; the intensity of the staining was monitored under a microscope during the staining procedures until red-orange (Alizarin red) or dark blue (Alcian blue) staining of the structures appeared. The duration of the staining was different for each set of samples. Alcian blue stained sections were then counterstained with nuclear red. Subsequently, the glass slides were dehydrated in absolute alcohol and mounted in mounting medium.

### Biomechanical testing

An evaluation of the mechanical properties of the rat Achilles tendons was carried out by destructive tensile tests in order to evaluate their strength and elastic characteristics. The experiments were performed using the MTS 858 Mini Bionix material testing system. The tendons were fixed between two metal clamps mounted in pneumatic clamp grips, ensuring a constant downforce and tough, stable attachment of the samples during cross-sectional changes. The grips were equipped with diamond-like apexes. The force impacts were recorded by a sensitive detector with a force range of 500 N (detector error max. 0.05%, e.g. 0.04 N error by the application of 80 N). The prepared tendons were stored in a physiological solution at 6°C. The mechanical testing was carried out at room temperature (22°C ±2°C).

The tendons were tested in a bone-to-muscle (BTM) arrangement, i.e. the tendons were removed with a part of the muscle on one side and with a part of the bone on the other side. This configuration enabled their secure attachment in the pneumatic clamp grips during the course of the test, assuming that the muscle tissue was inserted into liquid nitrogen for 5 sec before applying the load. While implementing this process, we also checked that the tendons were not frozen. The tendons were removed from the left and right limbs and were tested in pairs. In total, 42 tendons (21 pairs) were mechanically tested. After fixation, the tendon was pulled at a constant rate of 10.0 mm/min until failure. We recorded the applied force, the peak force and tendon elongation with a sampling frequency of 20 Hz.

The evaluation of the assessed data was aimed at discovering the strength and the elastic characteristics of the tested tendons. The stiffness of the tendons was evaluated from the course of the loading. The strength of the tendons was evaluated from the peak force at failure. Another important parameter for evaluating tendon stiffness was the ratio between the surgically intact (control) tendon and the surgically operated limb (treated or not treated with hMSCs) of the same animal. In this way, the effect of differences in the physiques of the animals could be minimized.

### Statistics

Due to the biological nature of the measured samples, statistically significant differences were evaluated by nonparametric methods (STATGRAPHICS Centurion XV, StatPoint, USA). The Kruskal-Wallis test was used for this purpose. The Mann–Whitney test was used as a post hoc test. A nonparametric analysis of variance was performed at the 95% confidence level (p = 0.05). Immunohistochemical and histological data are presented as mean ± standard deviation. The mean values for comparing the biomechanical data were calculated mainly as medians. The intervals for each median were calculated as the interquartile range (IQR). For comparative purposes, the mean values were also calculated as an arithmetical average, and their confidence intervals (CI) were calculated on the basis of t-statistics at the 95% confidence level (p = 0.05).

## Results

### Cell isolation and cultivation

**Figure 1:**
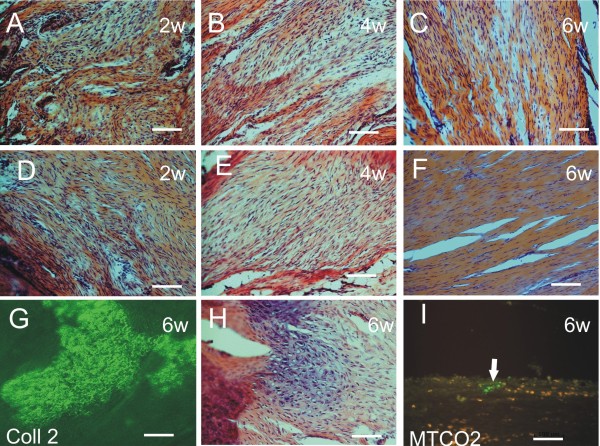
**Histology.** Hematoxylin-eosin sections show the improvement of tendon ultrastructure in control **(A-C)** and hMSC-treated **(D-F)** groups 2, 4 and 6 weeks after tendon injury. Better organization of the extracellular matrix was observed after hMSC treatment compared to the saline-treated tendons (Scores A-7, B-12, C-13, D-11, E-14, F 17). The formation of cartilage and bone was confirmed by collagen II staining, 6 weeks after injury **(G,H)**. Mesenchymal stromal cells survived at the site of the lesion as confirmed by MTCO2 staining **(I)**.

The GMP production of hMSCs was validated before the experiments. More than 90% of the cells simultaneously expressed CD105, CD73 and MHC I Class while being negative for CD45 and CD34; the preparations were sterile and without the presence of endotoxins or mycoplasma. The content of contaminating cells (i.e., cells displaying the positive expression of CD34, CD16, CD19, CD3, CD14 or CD79alpha) was below 5% in all samples. Cell viability was more than 95% before transplantation. Staining for MTCO2 (human mitochondria - Cytochrome c oxidase subunit II) confirmed the presence of transplanted hMSCs in the site of injury 2, 4 and 6 weeks after transplantation (Figure 1I).

### Health condition of the animals and gross inspection of the tendons

After animals awoke from the anaesthesia, no differences were observed between the saline and hMSC groups in the BBB motor performance test. The animals had a tendency to not use the lesioned limb and the stability of their trunk was altered; but after three days the animals fully recovered, and the injection of hMSCs/saline did not influence their motor performance. The wounds of 3 rats belonging to the saline-treated group needed to be resutured due to the animals’ activity. There were no gross failures detected by tendon anatomo-pathological observation in either group.

### Extracellular matrix

**Figure 2:**
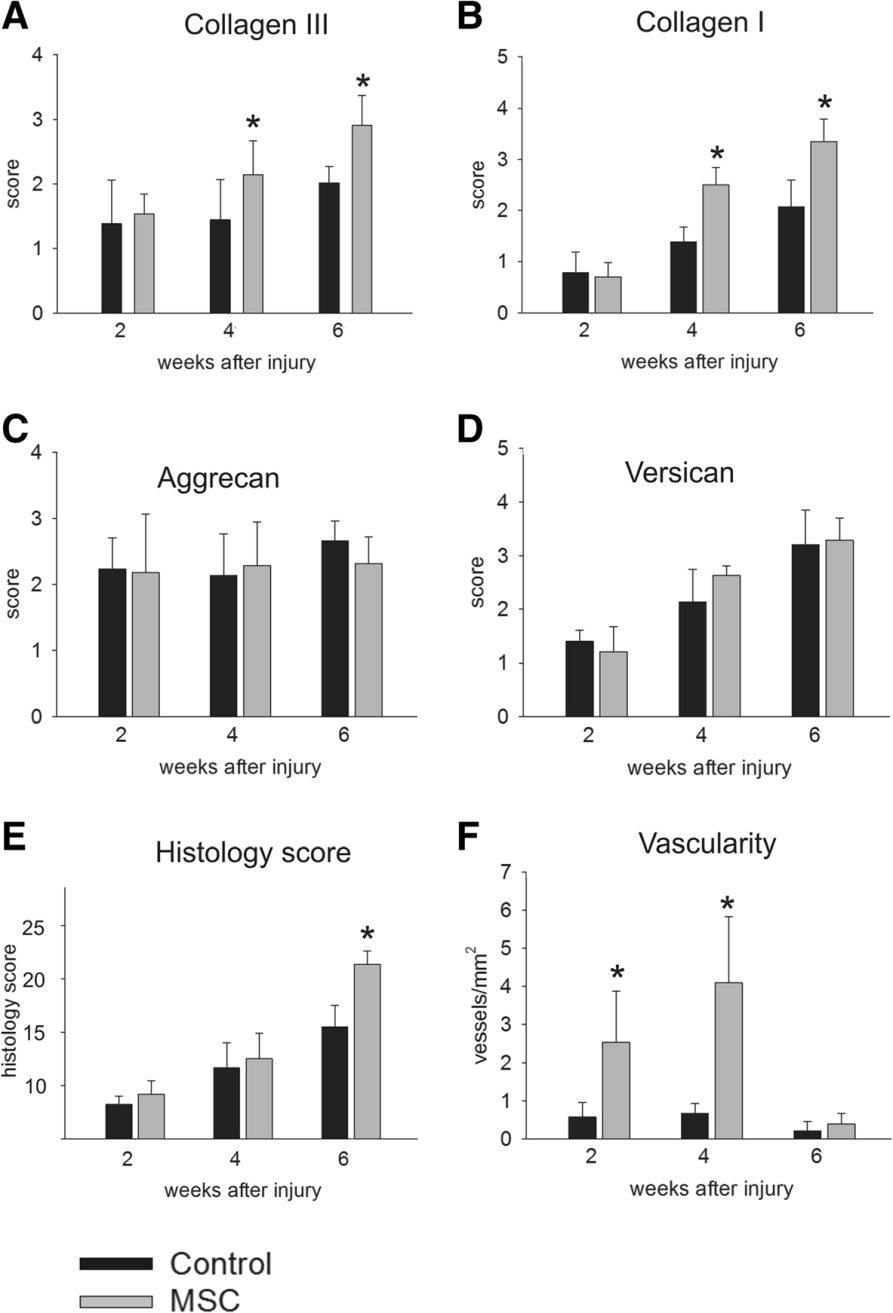
**Graphs. (A-D)** Graphes showing the time course of the semiquantitativly evaluated intensity of the extracellular matrix. **(A, B)** Intensity of collagen I and collagen III staining reached statistical significance 4 and 6 weeks after tendon injury (p < 0.05) **(C, D)** Aggrecan and versican semiquantitative grading showed no statistical differences between the hMSC-treated and control groups **(E)** Hematoxylin end eosin stained sections were evaluated for different criteria (linearity of their fibre structure, the shape of the tendon cells, the density of the tendon cells, inflammation, hemorrhage, and the thickness of the epitenon) and cumulative score are presented in the graph showing that hMSC-treated tendons had significantly higher scores 6 weeks after tendon injury **(F)**. The number of blood vessels per mm^2^ in the center of the lesion was calculated 2 and 6 weeks after tendon injury; neovascularization significantly increased in the hMSC-treated tendons.

Semiquantitative immunohistological analysis showed an increase of collagen III immunopositivity, and the scores of the hMSC-treated group were significantly higher 4 and 6 weeks after tendon injury (Figure 2A). Similarly, collagen I immunopositivity reached significantly higher scores 4 and 6 weeks after injury in the hMSC-treated group (Figure 2B). The aggrecan semiquantitative immunopositivity score remained stable during the 6 week survival period in both groups, and no statistically significant difference was found between the groups (Figure 2C). The versican semiquantitative score rose during the entire survival period in both groups, but differences between the groups did not reach statistical significance (Figure 2D).

### Histology score

Six weeks after tendon injury, the wounds were observed to be filled with connective tissue in both groups. The hMSC-treated group achieved a significantly better histology score (Figure 2E) thanks to their lower cellularity with the presence of more spindle-shaped cells, a denser tissue matrix, better vascularity and better organization of the collagen fibers (Figure 1D-F). P values less than 0.05 were considered significant.

### Angiogenesis

We observed excessive angiogenesis 14 days and 28 days after Achilles tendon injury in both groups. Forty-two days after injury, angiogenesis decreased in both the hMSC-treated and the saline-injected groups (Figure 2F). The number of RECA-positive vessels per mm^2^ was significantly higher in the hMSC-treated group 14 and 28 days post-injury, while no difference between the groups was observed 42 days after Achilles tendon injury (P < 0.05).

### Osteogenesis

We analysed osteogenesis within the damaged or treated tendons using Alizarin red stained sections. In 2 controls (20%) and 6 hMSC-treated animals (55%), there were deposits of calcified bone matrix of about 1 mm in the tendons. These bone deposits were surrounded by a thin layer of hyalinous cartilage (Figure 1H).

### Biomechanical testing

**Figure 3:**
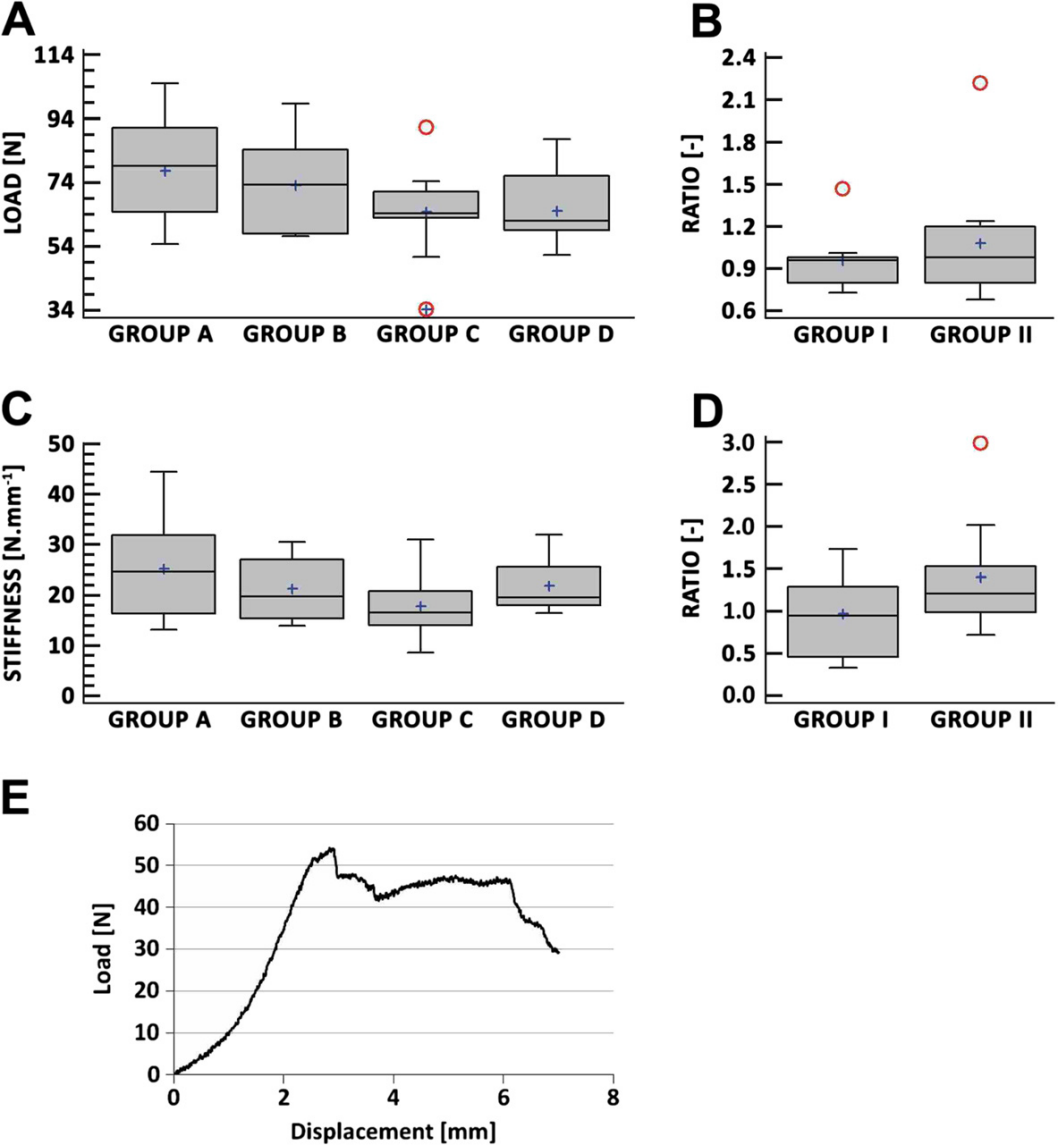
**Biomechanical testing. (A)** Box-and-Whiskers plot of the peak forces measured at tendon failure. **(B)** Ratio of the peak forces assessed at failure in intact and surgically treated tendons. **(C)** Box-and-Whiskers plot of the tendon stiffness. **(D)** Ratio of the stiffness of intact and surgically treated tendons. **(E)** A representative characteristic load–displacement curve from a tensile test of an Achilles tendon. Groups A and C contain intact (unoperated) tendons, group B contains surgically operated tendons without the application of mesenchymal stromal cells (hMSCs), group D contains surgically operated tendons with the application of hMSCs. Group I represents animals without hMSC treatment. Group II represents animals with hMSC treatment. The grey boxes indicate the interquartile range, the whiskers indicate the 10–90 percentile range, and the small red circles indicate values more than 1.5 times IQR above or below the boxes (○). Any points more than 3 times IQR above or below the box are indicated by circles with superimposed plus signs (⊕). A horizontal line is drawn at the median. The plus signs inside the boxes indicate the arithmetical averages.

The mechanically evaluated samples were divided into four groups. Group A contained intact tendons, while Group B contained tendons with a collagenase-induced lesion that was not treated by hMSC transplantation (operated). Group C contained intact tendons, and Group D contained lesioned tendons treated with an application of hMSCs. Groups A and B were taken from the same animal (left and right leg), similarly Groups C and D. The final mean peak force values assessed at tendon failure are listed in Table 3. The mean peak force values at failure of the operated tendons not treated with an application of hMSCs (Group B) were about the same as the values for the intact tendons (Group A); Mann–Whitney post hoc test (p = 0.05), for an illustration see Figure 3A. Similarly, the mean peak force values at failure of surgically operated tendons treated with an application of hMSCs (Group D) were about the same as the values for the intact tendons (Group C); Mann–Whitney post hoc test (p = 0.05). The ratios of the peak force at failure (B/A and D/C) were calculated for further verification of the influence of hMSC treatment (see Figure 3B). In Figure 3B, Group I represents the peak force ratios of hMSC-untreated defects, and Group II represents the peak force ratios of hMSC-treated defects. In this case as well, no statistically significant differences were found at the 95% confidence level.

**Table 3:** Biomechanical testing mean peak force values

	**F**_**max **_**[N]**			
	**Group A**	**Group B**	**Group C**	**Group D**
Median	79.2	73.4	64.4	62.1
IQR	[64.8; 91.2]	[58.1; 84.4]	[63.0; 71.2]	[59.1; 76.2]
Average	77.8	73.2	64.8	65.2
CI (*p = 0.05*)	[66.8; 88.7]	[63.7; 82.7]	[54.2; 75.2]	[56.7; 73.6]

The evaluation of elastic characteristics represents an assessment of Achilles tendon stiffness, which was calculated from a linear portion of the elastic phase of the load–displacement curve from the tensile test (see Figure 3E). The final mean tendon stiffness values are listed in Table 4. There were no statistically significant differences (p = 0.05) between the lesioned tendons not treated with an application of hMSCs (Group B) and the intact tendons (Group A), evaluated using the Mann–Whitney post hoc test. Similarly, there were no differences between the stiffness of the surgically operated tendons treated with an application of hMSCs (Group D) and the stiffness of the intact tendons (Group C), again using the Mann–Whitney post hoc test (p = 0.05); for an illustration see Figure 3C. The stiffness ratios (B/A and D/C) were calculated for further verification of the influence of hMSC treatment (see Figure 3D). In Figure 3D, Group I represents the stiffness ratios of hMSC-untreated defects, and Group II represents the stiffness ratios of hMSC-treated defects. In this case, too, no statistically significant differences could be found at the 95% confidence level. However, it should be pointed out that we found the highest confidence level for the presence of statistically significant differences among all groups in the comparison between Group D and Group C: statistically significant differences were found at the 90.3% confidence level. Statistically significant differences in the comparison between Group A and Group B were found at the 52% confidence level. We can therefore conclude that in this experimental design, the application of hMSCs could result in interesting effects – an increase in the stiffness of the tested tendons.

**Table 4:** Biomechanical testing stiffness values

	**Stiffness [N.mm**^**−1**^**]**			
	**Group A**	**Group B**	**Group C**	**Group D**
Median	24.5	19.8	16.6	19.6
IQR	[16.3; 31.9]	[15.4; 27.0]	[14.1; 20.9]	[18.0; 25.6]
Average	25.5	21.3	17.9	21.9
CI (*p = 0.05*)	[18.7; 31.7]	[17.3; 25.3]	[12.9; 22.9]	[18.0; 25.8]

## Discussion

Our main objective was to test the safety and effectiveness of human mesenchymal stromal cells transplanted 3 days after a collagenase tendon injury. No differentiation protocols were applied to the hMSCs, therefore their effect was dependent only on factors such as mechanical factors [10], paracrine signalling [11,12] and/or interleukins [13-15] present within the injured tendon. The inflammatory environment and the hypoxic conditions in the tendon also play an important role in cell behaviour, which cannot be completely replicated by in vitro experiments. This process can be compared to the differentiation protocol described by Lovati et al.; briefly, equine bone marrow MSCs were co-cultured with tendon tissue fragments in a transwell system [1]. In these experiments, 5% human platelet lysate was utilized as a culture supplement, as described by Warnke et al., who used 10% human platelet lysate for MSC propagation [16]. A similar effect on osteodifferentiation was previously described by Chevallier, Parsons and Prinse [17-19]; this effect can be related to the age of the platelet donors [20]. Other authors have cast doubts on the influence of platelet lysate on osteogenic differentiation [21-23]. Further experiments in this area are needed in order to utilize expanded hMSCs in therapeutic protocols for tendon healing because osteogenesis in tendons is undesirable and must be prevented.

The present results provide evidence that hMSCs enhance the healing processes in the rat after chemically induced tendon injuries. The production of collagen I, collagen III and versican increased during the 6 week survival period, and in the hMSC-treated group both types of collagen deposits reached a statistically significantly elevated level over the level seen in the saline-treated group. This production could be related to the direct synthesis of extracellular matrix proteins by the hMSCs during their maturation [24].

The structure and organization of the tendon tissue – expressed as a histology score – showed better organization of the extracellular matrix and lower cellularity in the hMSC-treated group. The level of aggrecan expression remained stable and was similar in the control and hMSC-treated tendons; other authors found that increased aggrecan mRNA expression is associated with painful tendinopathy [25]. As repeatedly described previously, hMSC transplantation had a strong influence on neovascularization [26]. In our experiments, 2 and 4 weeks after tendon injury the number of vessels in the hMSC-treated group was significantly higher at the site of injury. Versican expression has been detected in many tissues, including the brain, smooth muscles, cartilage and tendons [27]. Our semiquantitative immunohistochemical evaluation showed that the expression of versican non-significantly increased in the hMSC-treated group. Corps et al. [25] reported that a decreased level of versican was present in the tissue of painful and ruptured tendons. They concluded that decreased versican mRNA expression can contribute to alter matrix structure and function in chronic tendinopathy and ruptured tendons. Our semiquantitative immunoistochemical evaluation showed that the expression of versican protein was not influenced by hMSC implantation.

Collagen II positivity, Alcian blue and Alizarin red staining were used to investigate the formation of cartilage and bone at the site of injury. In three rats from the saline-treated group (30%) and eight rats from the hMSC-treated group (73%), the presence of newly formed cartilage at the site of injury was detected. It is probable that the cartilage was developing towards the bone that was present in 20% of controls and 55% of hMSC-treated tendons. The low vascularity of the tendon and the very dense suspension of hMSCs implanted into the lesion site established hypoxic conditions for the cells. hMSCs cultured under hypoxic conditions expressed higher levels of osteoblastic and adipocytic differentiation markers than normoxic controls [28]. Different protocols for increasing tenogenic differentiation and decreasing the risk of ectopic cartilage and bone formation using, e.g., growth and differentiation factors 5 and 6, have been developed [29,30]; however, before translation of the protocols to the clinical environment, their safety should be well documented.

Biomechanical testing of stiffness and load to failure did not result in differences between the groups. However, the stiffness of the tendons improved after the application of hMSC treatment at the 90% confidence level (27-85% taking into account the lower and upper quartiles, 18% taking into account the medians). From the point of view of the mechanical testing of biological materials, we can consider the 90% confidence level as a sufficient result; however, more *in vivo* experimental work is needed to clarify this. While lower than the widely used 95% and 99% confidence levels, the 90% confidence level in statistical hypothesis testing theory can indicate useful experimental effects. According to previous publications [31,32], we decided to test the biomechanical properties of the tendons 4 weeks after injury. However, as was shown in our results, the reorganization of the extracellular matrix and the healing processes still continued for another two weeks. Also, the vascularity of the tendon tissue was very high 4 weeks after injury and then decreased. Neovascularization is an important part of the healing process and ultimately enhances tendon regeneration, but it actually could change the mechanical properties of the tendon and affect the results between groups.

## Conclusions

Human multipotent mesenchymal stromal cells, when implanted into artificially induced tendinitis, promote neovascularization and tissue organization. The implantation may increase the stiffness of the tendons; however, more experiments are needed to clarify this point. The treatment procedure is safe, no tumor formation or excessive inflammatory reaction were detected; nonetheless, cartilage formation at the implantation site occurred in a few cases, and this should be taken into account when planning subsequent *in vivo* and clinical trials as an expected adverse event.

## Abbreviations

hMSCs: Human mesenchymal stromal cells; IQR: Interquartile range; CI: Confidence intervals; RECA: Anti-endothelial cell antibody; MTCO2: Anti-cytochrome c oxidase subunit II antibody; GMP: Good manufacturing practice; BBB: Basso-Beattie-Bresnahan motor performance test; H&E: Hematoxylin and eosin staining.

## Competing interests

The authors declare that they have no competing interests.

## Authors’ contributions

LMU and TA designed the experiments, performed the surgery of the rats, collected samples, evaluated the samples, performed histology and immunohistochemistry, analyzed the data and drafted the manuscript. RS and TS were involved in the biomechanical testing of the tendons, interpretation of the data and drafting of the manuscript. PL and PJ were involved in expanding and quality control of the mesenchymal stem cells, VH were involved in animal care and designing of experiments, ES interpreted data, designed study and critically revised the manuscript. JR was involved in statistical evaluation of immunohistochemistry, mesenchymal stem cells cultivation and prepared second and third revision of the manuscript. All authors approved the final manuscript.
